# Preoperative deep vein thrombosis in patients with cervical spondylotic myelopathy scheduled for spinal surgery

**DOI:** 10.1097/MD.0000000000005269

**Published:** 2016-11-04

**Authors:** Le Liu, Yan-Bin Liu, Jian-Min Sun, Hai-Feng Hou, Chen Liang, Tao Li, Heng-Tao Qi

**Affiliations:** a Department of Spine Surgery, Shandong Provincial Hospital, Shandong University, Jinan; b Department of Orthopaedic Surgery, People's Liberation Army 148 Hospital, Zibo; c Department of Orthopaedic Surgery, Liaocheng People's Hospital, Liaocheng; d Institute of Epidemiology, Taishan Medical University, Taian; e Department of Ultrasound, Shandong Medical Imaging Research Institute, Shandong University, Jinan, Shandong Province, P.R. China.

**Keywords:** cervical spondylotic myelopathy, deep vein thrombosis, preoperation

## Abstract

**Introduction:**

The prevalence of deep vein thrombosis (DVT) and its risk factors in patients with cervical spondylotic myelopathy (CSM) before spinal surgery are poorly understood. We investigated this association with a retrospective cross-sectional study.

**Patients concerns:**

The study cohort consisted of all consecutive patients with CSM who were scheduled for spinal surgery at our institution from 2013 to 2015. DVT was defined as an intraluminal filling defect in a lower extremity vein identified by Doppler ultrasonography.

**Outcomes:**

Of the 396 patients with CSM, 16 (4%) had DVT. Compared with patients without preoperative DVT, patients with preoperative DVT were older (62.75 ± 8.79 vs 53.03 ± 10.95 years, *P* = 0.001), had higher D-dimer concentrations (2.23 ± 4.15 vs 0.43 ± 0.90 mg/L, *P* *=* 0.04), had experienced longer duration of CSM (7.56 ± 7.08 vs 4.01 ± 6.37 months, *P* = 0.03), had lower Japanese Orthopaedic Association lower limb motor dysfunction scores (1.68 ± 1.25 vs 2.54 ± 0.91, *P* = 0.01), and had a history of ischemic cardiovascular events (33.3% vs 2.1%, *P* = 0.02). The area under the curve for the ability of D-dimer levels to predict DVT was 0.858 (95% confidence interval: 0.764–0.951; *P* < 0.0001). A D-dimer level of 0.54 mg/L detected DVT with a sensitivity and specificity of 87.5% and 83.2%, respectively. Abnormal D-dimer levels and ischemic cardiovascular events history were independent predictors of DVT.

**Conclusion:**

Patients with CSM who were scheduled for surgery often presented with preoperative DVT. Preoperative vascular screening should be considered for patients with CSM, especially for those who are older, have had longer duration of CSM, have poor lower limb mobility, and have a heart disease history. Inferior vena cava filter insertion and anticoagulation treatments should be considered for CSM patients with preoperative DVT.

## 1 Introduction

Cervical spondylotic myelopathy (CSM) refers to impaired function of the spinal cord that is caused by degenerative changes of the discs and facet joints in the cervical spine and results in spinal cord compression. It is the most common cause of myelopathy. It has been reported that 20% to 60% of patients with this disease will deteriorate neurologically over time without surgical intervention.^[1]^ Most patients with CSM are over 50 years of age. CSM is associated with tingling or numbness in the arms, fingers, and/or hands; arm muscle weakness; imbalance and other coordination problems; loss of fine motor skills; and pain and/or stiffness in the neck. While it can be treated conservatively, surgery is sometimes indicated. The estimated prevalence of surgically treated CSM is 1.6 per 100,000 inhabitants.^[2]^

Venous thromboembolism is a serious complication of spinal surgery. In particular, it is important to detect deep vein thrombosis (DVT) early because of the risk of pulmonary embolism (PE) and potentially fatal sequelae. Several studies have reported that the prevalence of venous thromboembolism (VTE) after spinal surgery ranges from 0.29% to 31%.^[3,4]^ Moreover, the overall rates of PE and death due to PE after spinal surgery are 1.38% and 0.34%, respectively.^[3–6]^ The soleal vein is the most common initial site of DVT. Ohgi et al reported that, of the thrombi found in patients who were suspected of having DVT distal to the popliteal vein, 61% were in the soleal vein; moreover, in the 6 patients who had symptomatic PE, thrombosis was in the soleal vein in all cases.^[7]^ However, the prevalence of and risk factors for DVT in patients with CSM after spinal surgery are still poorly understood.

The prevalence of DVT in patients with CSM before spinal surgery is also not known. It is of interest to identify risk factors for DVT in patients with CSM so that patients bearing these risk factors can be screened preoperatively for asymptomatic DVT. The present retrospective cross-sectional study was performed to determine the preoperative prevalence of DVT in patients with CSM who were scheduled to undergo spinal surgery to treat their CSM symptoms. The risk factors for DVT in this patient population were also assessed.

## 2 Methods

This retrospective cross-sectional study was approved by the Institutional Review Board of the Shandong Provincial Hospital in China. This tertiary referral hospital is affiliated with Shandong University. The need for informed consent from the patients was waived because of the retrospective nature of this study. The study was conducted according to the principles of the Declaration of Helsinki.

The study cohort consisted of all consecutive patients with CSM who were admitted to the Department of Spinal Surgery at the Shandong Province Hospital for scheduled spinal surgery between September 2013 and September 2015. Patients who did not undergo surgery were usually treated as outpatients and were excluded from this study due to incomplete data. All patients underwent lower limb Doppler ultrasonography (DUS) before surgery if their D-dimer levels were abnormal (≥0.55 mg/L) and/or they had symptoms of DVT (pain and swelling of the lower limb). Patients underwent ultrasonography of the popliteal and/or calf vein proximal to the femoral and pelvic veins, as well as the inferior vena cava (IVC). DVT was defined as the presence of an intraluminal filling defect in a lower extremity vein detected by DUS. Patients were included in the study if their medical records were complete with the following recorded information: sex, age, comorbidities (i.e., ischemic cardiovascular events, hypertension, diabetes, and/or dyslipidemia), lower limb DUS results, D-dimer concentrations, duration of CSM, and Japanese Orthopaedic Association (JOA) lower limb motor dysfunction scores.^[8]^ Patients were excluded if they had risk factors that could influence the JOA limb motor dysfunction scores, including cervical radiculopathy, trauma, use of anticoagulants (e.g., warfarin and aspirin) in the week before hospital admission, varicose veins combined with thoracic spinal stenosis or thoracic ossification of the ligamentum flavum, lumbar disc herniation, and/or physical dysfunction caused by stroke, cerebral palsy, brain surgery, or congenital disease.

### 2.1 Statistical analysis

All results for continuous variables were expressed as mean ± standard deviation; all data satisfied the criteria for normality and homogeneity of variance. Categorical data results were presented as numbers and percentages. Results for patients with and without preoperative DVT were compared using independent *t* tests and Chi-squared tests. Receiver-operating characteristic curve (ROC) analysis was used to identify the D-dimer level that best predicted DVT. Correlations between variables were analyzed by calculating Pearson correlation coefficients. Multivariate analysis was used to identify independent risk factors for preoperative DVT in patients with CSM. On 2-tailed tests, *P* values of <0.05 were regarded as statistically significant. All statistical analyses, except the ROC analysis, were performed with SPSS 16.0 (IBM SPSS, Chicago, IL). ROC analysis was performed with MedCalc 13.0 (MedCalc Software, Ostend, Belgium).

## 3 Results

Of the 465 patients with CSM who were scheduled for cervical spine surgery during the study period, 6 (1.29%) were excluded because their medical records were incomplete. Another 8 (1.72%) and 15 (3.22%) patients were excluded because they had cervical radiculopathy and trauma, respectively. Moreover, 7 (1.50%) had taken anticoagulants the week before hospital admission, 1 (0.21%) had varicose veins combined with thoracic spinal stenosis or thoracic ossification of the ligamentum flavum, 20 (4.30%) had lumbar disc herniation, and 12 (2.58%) had physical dysfunction caused by stroke, cerebral palsy, brain surgery, or congenital low limb disease, leading to exclusion from the study. A total of 396 patients were included in the study.

In total, 16 (4%) patients were found to have preoperative DVT. Compared with the 380 (96%) patients without preoperative DVT, patients with DVT were significantly older (62.75 ± 8.79 vs 53.03 ± 10.95 years old; *P* = 0.001), had higher D-dimer levels (2.23 ± 4.15 vs 0.43 ± 0.90 mg/L; *P* = 0.04), had experienced a longer duration of CSM (7.56 ± 7.08 vs 4.01 ± 6.37 months; *P* = 0.03), and had lower JOA scores (1.68 ± 1.25 vs 2.54 ± 0.91; *P* = 0.01) (Table 1). Twelve of the 16 cases of preoperative DVT were soleal vein DVT. The remaining 4 cases were wide range DVT (n = 1), popliteal vein DVT (n = 1), and posterior tibial vein combined with soleal vein DVT (n = 2). There were no cases of PE. No postoperative DVT cases occurred; 1 patient suffered from an IVC thrombus after IVC filter insertion. Patients were usually discharged from the hospital within 1 week after operation.

**Table 1:**
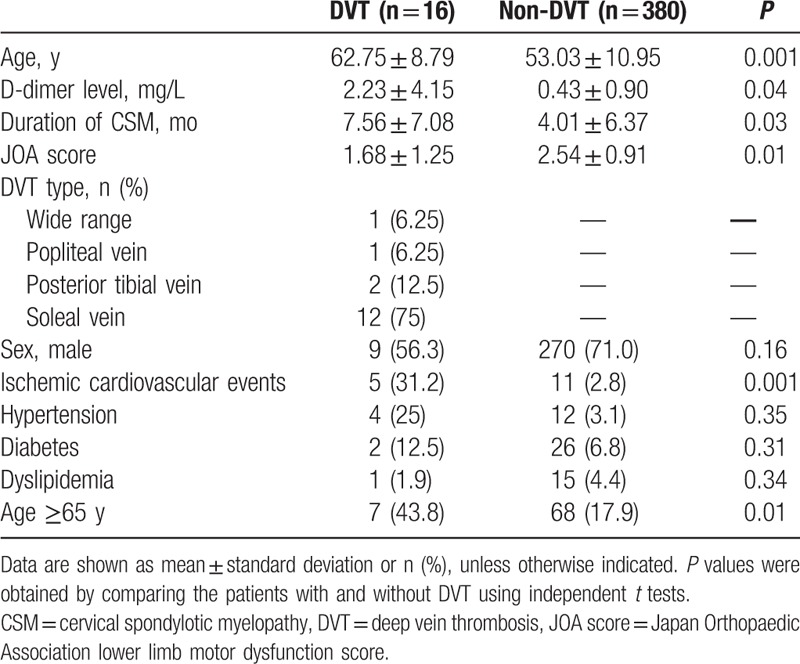
Demographic and clinical characteristics of the patients.

Chi-squared tests showed that, compared with the patients without DVT, patients with DVT were significantly more likely to have ischemic cardiovascular events (31.2% vs 2.8%; *P* = 0.001) and to be older than 64 years of age (43.8% vs 17.9%; *P* *=* 0.01) (Table 1). Sex, hypertension, diabetes, and dyslipidemia were not associated with preoperative DVT in patients with CSM.

**Figure 1:**
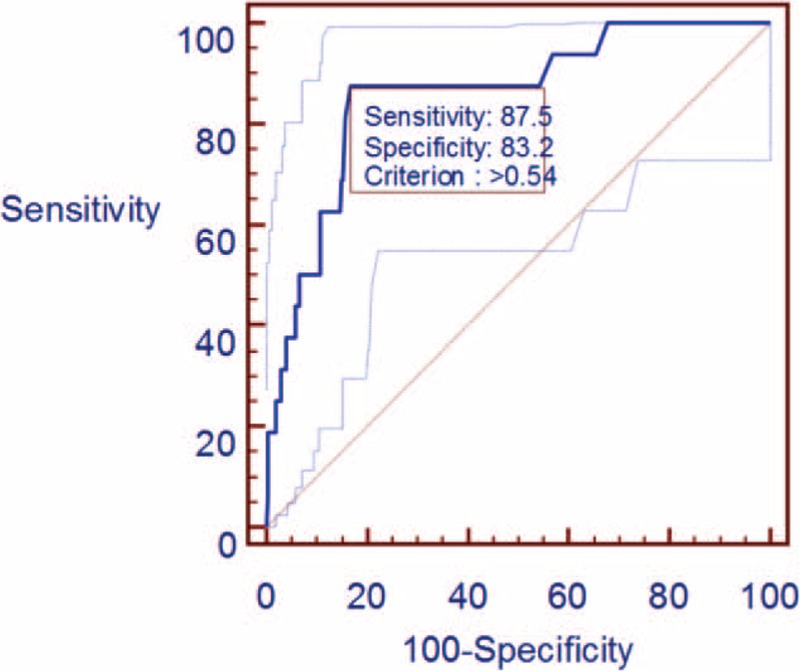
Receiver-operating characteristic (ROC) curve analysis of the sensitivity and specificity with which D-dimer testing detects preoperative deep vein thrombosis in patients with cervical spondylotic myelopathy.

Analysis of the ROC curve for D-dimer levels indicated that the area under the curve was 0.858 [95% confidence interval (CI): 0.819–0.891; *P* < 0.0001] (Fig. 1, Table 2). The D-dimer value that best predicted DVT in patients with CSM was >0.54 mg/L; this value predicted DVT in our cohort with a sensitivity and specificity of 87.5% (95% CI: 61.7–98.4) and 83.2% (95% CI: 79.0–86.8), respectively.

**Table 2:**
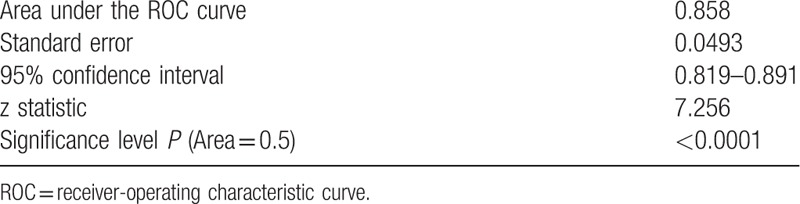
Area under the receiver-operating characteristic curve.

Correlation analysis results for the cohort showed that D-dimer levels were negatively correlated with JOA scores (r = -0.174, *p* < 0.01) and positively correlated with age (r = 0.253, *P* < 0.01). In addition, JOA scores were negatively correlated with age (r = -0.237, *P* < 0.01) (Table 3).

**Table 3:**
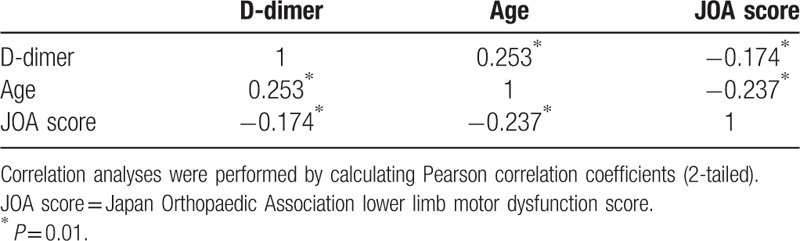
Correlations between D-dimer level, age, and Japan Orthopaedic Association lower limb motor dysfunction score in preoperative patients with cervical spondylotic myelopathy (n *=* 396).

Multivariate analysis showed that D-dimer levels and ischemic cardiovascular events history were independent risk factors for preoperative DVT in patients with CSM (Table 4).

**Table 4:**
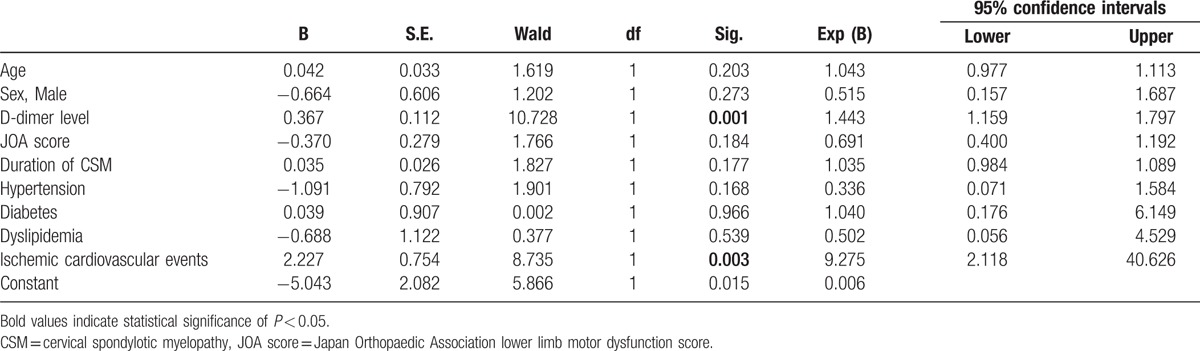
Multivariate analysis to identify factors independently associated with a higher risk of preoperative deep vein thrombosis in patients with cervical spondylotic myelopathy.

## 4 Discussion

The rate of and risk factors for DVT in patients with CSM before or after spinal surgery have remained relatively unknown. The current study focused on the prevalence of preoperative DVT in patients with CSM who were scheduled to undergo spinal surgery. We also assessed risk factors for preoperative DVT in patients with CSM to identify patients who should undergo vascular evaluation to exclude the presence of asymptomatic DVT.

This is the first study to estimate the prevalence of DVT in patients with CSM who were scheduled to undergo spinal surgery to treat CSM symptoms. The prevalence of DVT was 4%. By contrast, Masuda et al^[9]^ reported that the prevalence of DVT in 268 patients with traumatic cervical spinal injuries was 10.4% in a 4-week study. Results from another study showed a 1-year incidence of DVT of 11.2% in amyotrophic lateral sclerosis (ALS) patients with leg-onset ALS or significant leg weakness.^[10]^

There were no postoperative DVT cases in our study, except 1 IVC thrombus after IVC filter insertion. We can infer that the single case of postoperative DVT may have occurred preoperatively and was overlooked until postoperative detection. It is still considered more important to screen for DVT preoperatively.

Our study showed that age might be a risk factor for DVT in CSM; patients with preoperative DVT were significantly older than those without DVT (62.7 vs 53.0 years, *P* *=* 0.001). Another possible risk factor for DVT in patients with CSM was poor mobility of the lower extremities. Patients with preoperative DVT had significantly poorer mobility of the lower extremities than patients without DVT (JOA scores: 1.68 and 2.54, respectively; *P* = 0.03). Notably, we found a negative correlation between age and JOA score (r = -0.237, *P* < 0.01). This suggests that older patients with CSM may be more prone to DVT than younger patients because their mobility is more restricted. A longer duration with CSM was also associated with DVT in CSM; patients with preoperative DVT had CSM for a significantly longer duration than patients without DVT (7.56 vs 4.0 months; *P* = 0.03). These observations are consistent with those of a study by Ohmori et al, ^[11]^ which found that 34.8% of patients with severe motor disabilities had DVT; this may have reflected their restricted mobility and tendency to be bedridden for long periods. Moreover, a study by Hamidi and Riazi^[12]^ showed that postsurgical recumbence duration was an independent risk factor for VTE after spinal surgery. However, age, CSM duration, and JOA score were not independent risk factors for preoperative DVT in our patients in multivariate analysis.

DVT in CSM was also associated with ischemic cardiovascular events; 31.2% of patients with DVT had ischemic cardiovascular events compared with 2.8% of the patients without DVT (*P* *=* 0.001). Multivariate analysis found this covariate to be an independent risk factor of DVT in CSM. The pathophysiology of venous thrombosis involves blood stasis, hypercoagulability, and endothelial damage. A previous study showed that DVT patients more frequently have impaired flow-mediated dilation (FMD), recognized as an indicator of arterial endothelial dysfunction and a marker for increased cardiovascular risk.^[13]^

The D-dimer assay can effectively predict the occurrence of DVT, and D-dimer levels of ≥0.5 mg/L are considered a risk factor for DVT after spinal surgery.^[14]^ A number of factors influence the sensitivity and specificity with which D-dimer tests predict DVT, including the extent of thrombosis and fibrinolytic activity, the duration of symptoms, age, surgical procedures, treatment with anticoagulants, and previous VTE.^[15]^ Nevertheless, 2 studies showed that in patients with suspected thromboembolism, D-dimer testing detected DVT with a sensitivity and negative predictive value of 100% and 100%, respectively^[16,17]^; the lower bound of the 95% CI for both the sensitivity and negative predictive value exceeded 95%.^[17]^ In our study, the D-dimer cut-off of >0.54 mg/L predicted DVT in our population of CSM patients with a sensitivity of 87.5% (95% CI: 61.7–98.4) and a specificity of 83.2% (95% CI: 79.0–86.8). The overall accuracy of the D-dimer test was 0.858 (95% CI: 0.819–0.891). Thus, D-dimer testing identified patients with DVT with relatively high sensitivity and specificity. Interestingly, we found that D-dimer levels correlated negatively with JOA scores (r = -0.174, *P* < 0.01) and positively with age (r = -0.253, *P* < 0.01). This can be explained by the fact that D-dimer concentration increases with age.^[18]^ In multivariate analysis, D-dimer levels were an independent risk factor for DVT in our population. This suggests that suspicion of preoperative DVT in CSM should arise when D-dimer level exceeds 0.54 mg/L, especially when the patients are older or have low JOA scores.

IVC filters have often been used to prevent fatal PE; ^[9,14]^ however, indications for IVC filters are controversial and sometimes excessive. Clear indications for venous interruption in patients with DVT are anticoagulant-induced bleeding or anticipation of hemorrhagic complications in patients with a predisposing lesion and anticoagulation failure.^[15]^ In our study, 2 patients had proximal DVT, 1 above the popliteal vein and the other above the popliteal vein combined with the soleal vein. As proximal DVT is more likely to cause fatal PE than distal DVT, especially during operation, both patients underwent IVC insertion. One patient suffered from IVC thrombus as a complication related to IVC insertion. In the remaining 14 cases with DVT, the thrombi were resolved by treatment with low molecular weight heparin. All of these patients underwent spinal surgery 1 month later. DUS at that time showed resolution of the thrombosis in the lower limb vein.

This study had a number of limitations. The retrospective design is prone to selection and information bias. The number of patients with DVT was relatively small, which may have limited the power of the study to detect associations, and larger scale prospective studies that test our observations are warranted. The specificity and sensitivity for detection or exclusion of DVT via D-dimer testing is not high in our study. The postoperative follow-up period was short and data on long-term outcomes were limited, which may limit postoperative DVT detecting. So, the relationship between preoperative and postoperative DVT was not explained.

In summary, the prevalence of preoperative DVT in patients with CSM was relatively high (4%). Risk factors for asymptomatic DVT in patients with CSM were abnormal D-dimer levels and a history of ischemic cardiovascular events. Clinicians should consider vascular evaluation for patients with CSM when they have these risk factors. Poor lower limb JOA scores, a longer duration with CSM, and older age are also risk factors for asymptomatic DVT. As a screening test for DVT, D-dimer evaluating is cost-efficient and simple to administer, and could then be followed up with additional investigations such as ultrasonography or venography. Treatments such as insertion of an IVC filter and anticoagulation should be considered for patients with CSM who present with preoperative DVT.

## Acknowledgments

The authors would like to thank Mr. Yanbin Liu and Mr. Chen Liang for their assistance with data collection. Mr. Haifeng Hou made a significant contribution to the statistical analyses. We thank the anonymous reviewers for their useful comments, which have greatly improved the manuscript. We would like to thank Editage (www.editage.com) for English language editing.
